# Comparison of MicroRNA Deep Sequencing of Matched Formalin-Fixed Paraffin-Embedded and Fresh Frozen Cancer Tissues

**DOI:** 10.1371/journal.pone.0064393

**Published:** 2013-05-16

**Authors:** Wei Meng, Joseph P. McElroy, Stefano Volinia, Jeff Palatini, Sarah Warner, Leona W. Ayers, Kamalakannan Palanichamy, Arnab Chakravarti, Tim Lautenschlaeger

**Affiliations:** 1 Department of Radiation Oncology, Wexner Medical Center, Arthur G. James Comprehensive Cancer Center and Richard L. Solove Research Institute, The Ohio State University, Columbus, Ohio, United States of America; 2 Center for Biostatistics, The Ohio State University, Columbus, Ohio, United States of America; 3 Department of Molecular Virology, Immunology and Medical Genetics, The Ohio State University, Columbus, Ohio, United States of America; 4 Microarray Shared Resource, Arthur G. James Comprehensive Cancer Center and Richard L. Solove Research Institute, The Ohio State University, Columbus, Ohio, United States of America; 5 Department of Pathology, College of Medicine, The Ohio State University, Columbus, Ohio, United States of America; University of Connecticut Health Center, United States of America

## Abstract

MicroRNAs regulate several aspects of tumorigenesis and cancer progression. Most cancer tissues are archived formalin-fixed and paraffin-embedded (FFPE). While microRNAs are a more stable form of RNA thought to withstand FFPE-processing and degradation there is only limited evidence for the latter assumption. We examined whether microRNA profiling can be successfully conducted on FFPE cancer tissues using SOLiD ligation based sequencing. Tissue storage times (2–9 years) appeared to not affect the number of detected microRNAs in FFPE samples compared to matched frozen samples (paired t-test p>0.7). Correlations of microRNA expression values were very high across microRNAs in a given sample (Pearson’s r = 0.71–0.95). Higher variance of expression values among samples was associated with higher correlation coefficients between FFPE and frozen tissues. One of the FFPE samples in this study was degraded for unknown reasons with a peak read length of 17 nucleotides compared to 21 in all other samples. The number of detected microRNAs in this sample was within the range of microRNAs detected in all other samples. Ligation-based microRNA deep sequencing on FFPE cancer tissues is feasible and RNA degradation to the degree observed in our study appears to not affect the number of microRNAs that can be quantified.

## Introduction

MicroRNAs are noncoding small RNAs with a length of 22 to 26 nucleotides (nt). MicroRNAs result in degradation of complementary messenger RNA in many species. MicroRNAs play an important role in cell development, cell death, cell proliferation [1], apoptosis [2] and angiogenesis [3]. The pioneer bead-based flow cytometric microRNA expression profiling method revealed that microRNA profiles reflect the developmental lineage and differentiation of tumors [4]. Normal tissues and cancer tissues were shown to have distinctive expression profiles and microRNA profiles can describe microRNA-driven pathways in solid tumors [5].

The current methods for microRNA quantification are: real-time reverse transcription-PCR [6], [7], microarray [8], Nanostring, and next generation sequencing [9]–[11]. Especially, the newly developed next generation sequencing technologies have the potential to discover novel microRNAs and other small RNAs. Most tissues processed in the health care setting will undergo formalin-fixation and paraffin-embedding (FFPE). Most clinical assays performed in pathology laboratories are optimized for FFPE tissues. FFPE tissues are typically stored at room temperature. Formalin preserves tissue samples by creating cross-linking between proteins, DNA and RNA. DNA extracted from FFPE tissue has been shown to be useful for copy number analysis and mutation analysis on at least one platform in some settings [12]. However, the chemical modification between macromolecules and RNAs caused by formalin fixation can accelerate the degradation of RNA [13] [14]. The stability of microRNAs is generally much more robust than messenger RNA [15]. Next generation sequencing based microRNA expression analysis has been developed on several platforms, including Roche/454 platform, Illumina’s Genome Analyzer and ABI's SOLiD platform [16] [17], and different commercial protocols for miRNA library preparation have been developed by the three companies [18]. There is currently only limited evidence available that microRNA sequencing results are reliable and valid. Ma et al. demonstrated the feasibility of miRNA profiling based on Sanger sequencing in 10-year-old archived tissue [19]. To our knowledge there are only two peer-reviewed studies published that compared microRNA sequencing results of matched frozen and FFPE specimens using the Illumina platform [9], [20]. The objective of this study was to determine if FFPE samples can be successfully characterized by deep miRNA sequencing. To this end we analyzed matched frozen and FFPE tissues of different histologies for which detailed processing and storage information was available.

## Materials and Methods

### Ethics Statement

Human malignant tissue samples procured during period 2003–2009 were obtained from the Cooperative Human Tissue Network (CHTN/NCI), Midwestern Division, which is funded by the National Cancer Institute, based on an internal review board (IRB) approved research protocol (Ohio State University Human Subjects Protocol - 2011E0377). Other Investigators may have received specimens from the same subjects.

### Tissue specimens

Eight paired human malignant tissue samples were received. Remnant surgical tissues were taken after diagnostic samples were secured from patients (five males and four females; median age 58 years, range 39–78 years) with breast invasive ductal carcinoma (2), renal clear cell carcinoma (2), lung adenocarcinoma (1), prostate adenocarcinoma (1), metastatic melanoma (1), and sarcoma of thigh (1). Surgical specimens were transported from the operating rooms to Tissue Procurement, examined under a pathologist’s supervision and preserved within 5 to 57 recorded minutes. Paired tissue samples selected for research were either immediately snap frozen in liquid nitrogen and placed in a –80°C freezer for monitored storage or fixed in 10% buffered formalin for up to 24 hours and then processed into a paraffin embedded block and stored at room temperature. When the research samples were received from CHTN, the coded samples were accompanied by a coded final pathology report and a quality assessment report verifying collection of tumor tissue. The specimens have been reviewed by a pathologist and 4 cases had 100% tumor, 3 cases 90–95% and 1 case 50–60% tumor, and matched FFPE and frozen samples were generally comparable in tumor content and size. The clinicopathologic features of the cases used for microRNA sequencing and expression analysis are listed in table 1.

**Table 1:** The clinicopathologic features of the eight cases used for microRNA sequencing and expression analysis.

Case	Age	Gender	Location	Pathological diagnosis	Time of Collection(MM-YY)	Time to cryopreservation(min)
1	75	F	Kidney	Clear cell carcinoma	Oct-03	30
2	68	F	Breast	Invasive ductal carcinoma	Sep-05	5
3	67	F	Kidney	Clear cell carcinoma	Feb-08	35
4	43	M	Pelvis	Metastatic melanoma	Feb-10	30
5	39	M	Thigh	Sarcoma	Jun-07	NA
6	50	F	Breast	Invasive ductal carcinoma	May-08	24
7	43	M	Lung	Adenocarcinoma	Aug-09	57
8	78	M	Prostate	Adenocarcinoma	Apr-06	30

### RNA Isolation, quantitation, and quality Assessment

RNA from FFPE samples were extracted using Recover All Total Nucleic Acid Isolation Kit (Life Technologies Corporation, Carlsbad, CA). Briefly, 5–10 mg samples were sliced from paraffin blocks and deparaffinizated by xylene at 50°C, followed by 100% ethanol wash. The air-dry tissue samples were digested by proteinase K for 24 hrs in a microtube shaking incubator set at 50°C. The digested samples were mixed with appropriate volume of isolation additive and 100% ethanol. After passing the mixture through the filter cartridge, the DNA and RNA were retained on the filter. The DNA was removed by on-filter DNase digestion. The RNA was purified by washing buffer and eluted with nuclease-free water.

The microRNA samples from the fresh frozen samples were extracted using PureLink™ miRNA Isolation Kit (Life Technologies Corporation, Carlsbad, CA). Briefly, 5 mg fresh frozen tissue samples were mixed with 300 µl Binding Buffer (L3) and homogenized using tissue homogenizer. The homogenized samples were centrifuged, and supernatant were mixed with 300 µl 70% ethanol. The solution containing the RNA was purified by two round of spin columns cleanup. The RNA was eluted with 50–100 µl sterile RNase-free water.

### MicroRNA sequencing

Small RNAs from FFPE and fresh frozen samples were prepared for SOLiD sequencing as follows: the total RNA samples were processed by the flashPAGE fractionator (Ambion) and flashPAGE Clean-Up Kit (Ambion). The enriched small RNA was then processed according to the SOLiD Small RNA Expression Kit protocol (Applied Biosystems). The purified small RNAs were ligated with 5' and 3' adapter mix using RNA ligase. The ligated products (40–60 bases in length) were reverse transcribed and purified on Novex 10% TBE-Urea gel. Subsequently, 15–18 cycles of PCR were performed by amplifying the purified cDNA with barcoded PCR primer sets provided in the kit, which differed by a unique 6-nucleotide sequence. The amplified products were loaded on Novex 6% TBE gel (Invitrogen) and the gel bands containing 110 to 130 bp fragments were excised. The amplified products were purfied from excised gel band, amplified by emulsion PCR, then loaded on Applied Biosystems SOLiD 4 next generation high throughput sequencing system for data acquisition. The quality of the samples and libraries were verified on the Agilent Bioanalyzer [21], [22]. The raw SOLiD sequencing data were uploaded to the NCBI's Gene Expression Omnibus and are accessible through GEO Series accession number GSE45740 (http://www.ncbi.nlm.nih.gov/geo/query/acc.cgi?acc=GSE45740).

### RT-qPCR for microRNA validation

The RNA from fresh frozen and FFPE tissues were validated by real-time (RT) quantitative PCR. microRNA expression was normalized to the small nuclear RNA U6 using TaqMan MicroRNA Assay kits (Applied Biosystems, Darmstadt, Germany). A 30 ng sample of RNA was processed by the TaqMan MicroRNA Reverse Transcription kit (Applied Biosystems, Darmstadt, Germany). In brief, 5 µl RNA was mixed with 1 mmol/l of each deoxyribonucleotide triphosphate, 50 units of Multiscribe Reverse Transcriptase, 5× reaction buffers, 4 units RNase inhibitor, and 5× gene-specific RT primers mix in a final reaction volume of 15 µl. Reactions were then incubated at 16°C for 30 min, 42°C for 30 min, 85°C for 15 min with a final hold at 4°C. Afterward, 15 µl of the cDNA solution was diluted by nuclease-free water to a final volume of 100 µl. Quantitive PCR was run on a 7900 HT PCR machine with denaturation step at 95°C for 10 minutes, followed by 40 cycles of a denaturation step at 95°C for 15 seconds, and an annealing/elongation step at 60°C for 60 seconds. Fold change for microRNA in tissue samples was then calculated using the equation 2-ΔCt test/2-ΔCt control.

### Data analysis

The linker sequences of small RNA libraries were removed using cutadapt software (http://code.google.com/p/cutadapt/) under default parameters. The length distribution of microRNA was constructed by plotting reads counts against 0–30 nucleotide lengths. The small RNA Analysis Pipeline Tool from Applied Biosystems was used with the following parameters to include only sufficiently high quality reads in our study: 1) Minimal average read quality threshold was 20 (The QV value is calculated using a phred like score q = –10× log10 (p) where q is the quality value and (p) is the predicted probability that the color call is incorrect.), 2) to be stringent we allowed only 1 mismatch between reads and miRBase version 16 hits for each counted read 3) the minimal length of aligned reads had to be 17 bases. Data were normalized as reads per million (RPM). Sequences with expression value below 5 RPMs were considered not detected for the correlation between frozen and FFPE analysis part.

## Results

### Effects of FFPE sample degradation: microRNA read length distribution

**Figure 1:**
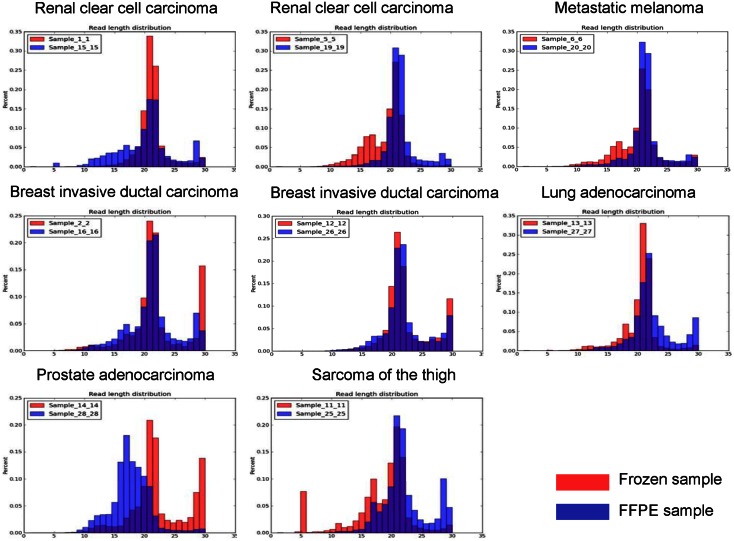
Sequence reads length distribution among eight paired fresh frozen and FFPE samples.

Formalin fixation and long storage times are known to result in RNA degradation [23]. This degradation results in a shorter average RNA length [24]. To identify possible degradation effects of microRNAs, we analyzed the read length distribution of matched fresh frozen and FFPE cancer tissue samples. After the 3’ adaptor sequences of each read were trimmed, sequence reads length distribution was examined (figure 1). Read lengths were observed to be centered at 21 nt in all frozen samples and all but one FFPE samples. There was one FFPE sample whose peak microRNA length was found at 17 nt, indicating significant degradation. We then carefully examined the small RNA classification profiles and correlation of microRNA expression values of fresh frozen and FFPE samples. We found that there are negligible or no effects on those analyses. Storage times or preparation methods were not different between this degraded FFPE sample and the other samples analyzed in this study. Also, the proportion of small RNAs in the 20–22 nt range was lower for several cases in the FFPE compared to the matched frozen specimens (one renal cell carcinoma case, the lung adenocarcinoma case, and the prostate cancer case). This is also likely due to degradation in FFPE samples, although small variations during the library preparation could partially contribute to the observed differences.

### Effects of FFPE sample degradation: storage time

**Figure 2:**
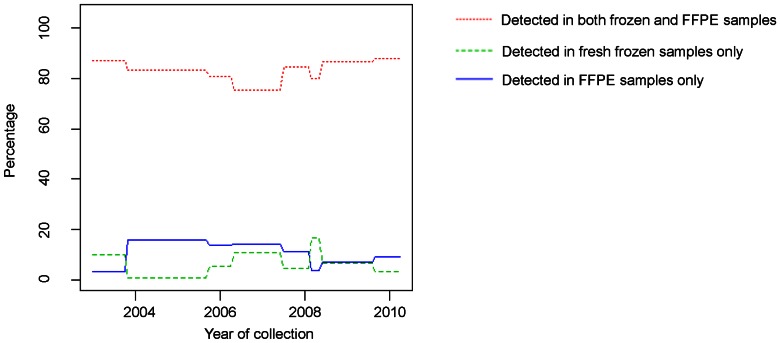
Percentages of microRNA reads detected over the years of specimen collection.

The storage time of the samples ranged from 2 to 9 years. 624 micoRNAs and their precursors were identified in the 8 pairs of tissue samples. Some microRNAs could not be detected in either fresh frozen or FFPE samples. Figure 2 shows the percentages of microRNAs that were detected in both fresh frozen and FFPE samples, only in fresh frozen samples and only in FFPE samples over the year of specimen collection. There were no storage-time-dependent changes in those percentages, indicating that there is no significant association between storage time and the number of microRNAs present during this 9 year time window.

**Figure 3:**
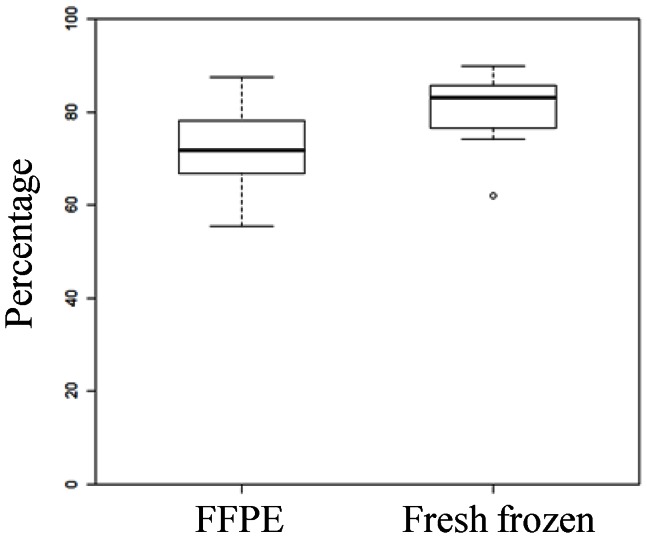
Percentage of microRNA reads among total reads.

**Figure 4:**
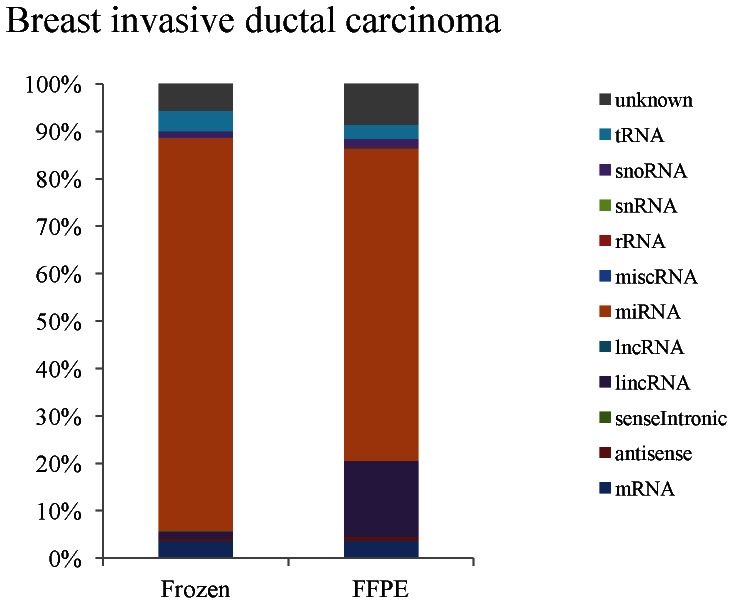
The classification of small RNA populations in fresh frozen samples and FFPE samples.

We next examined the percentage of microRNA reads among total reads (figure 3) to understand if the purity of microRNAs was reduced in the FFPE samples. Approximately 70% and 85% of reads in FFPE and frozen samples, respectively, were identified as microRNA sequences. A possible reason for this difference could be the presence of fragments of lncRNAs and mRNAs in the fraction of RNA smaller than 40 nucleotides (nt) in FFPE tissues (figure 4).

### Effects of FFPE sample degradation: classification of small RNAs

To better understand if the difference in the percentage of small RNA sequences among all reads between frozen and FFPE tissue data we examined the distribution of RNA classes in one of the breast cancer sample. There were a total of 11563414 and 10273837 sequencing reads, in the two examined matched samples and the reads were mapped to the human genome (hg19, National Center of biotechnology information building) using custom Python scripts. The reads were classified in different RNA groups according to the EnsEMBL database. The majority of small RNAs in both fresh frozen and FFPE libraries were microRNAs (figure 4). Comparing data from matched fresh frozen samples to data from FFPE samples there were lincRNA and sequences of unknown classes in data obtained from FFPE samples. Given that only RNAs smaller than 40 nt were extracted and processed for sequencing this suggests that there is fragmentation of lincRNA and other long RNA in FFPE tissues, which are longer than 40 nt.

**Figure 5:**
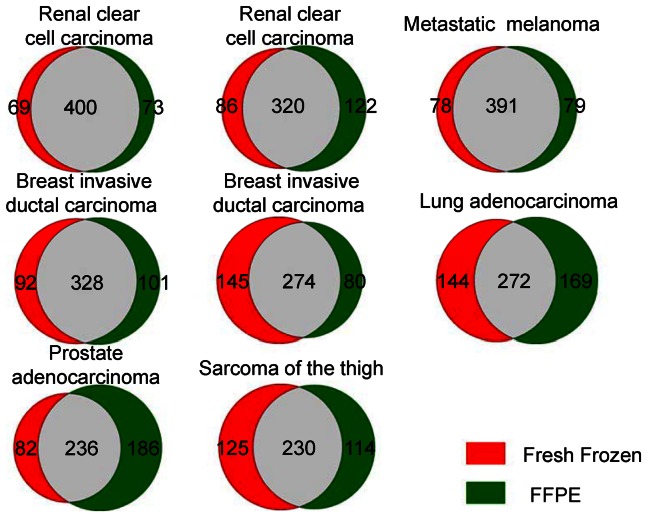
The number of microRNAs that have more than a 2 fold expression change between matched fresh frozen and FFPE samples.

A total of 469 to 548 (mean 519) different microRNAs and their precursors were identified in both fresh frozen and FFPE samples. We determined the percentages of microRNAs that have more than a 2 fold difference of expression between matched fresh frozen and FFPE samples (figure 5). Among the different cases, 69 to 145 microRNA transcripts were more than 2 fold overexpressed in fresh frozen samples. 73 to 186 microRNA transcripts were more than 2 fold overexpressed in FFPE tissues (figure 5).

### Correlation of microRNA expression values of matched FFPE and frozen samples

**Figure 6:**
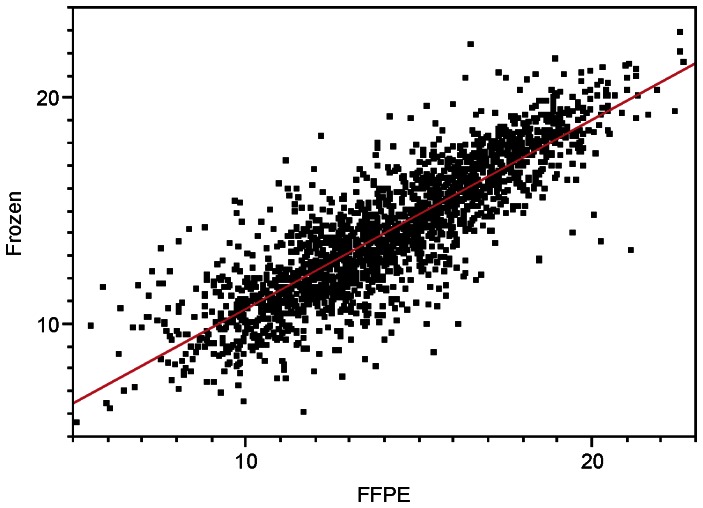
Regression of Frozen miR values onto FFPE miR values across all individuals and miRs. Only the 250 miRs with no missing data were used.

**Figure 7:**
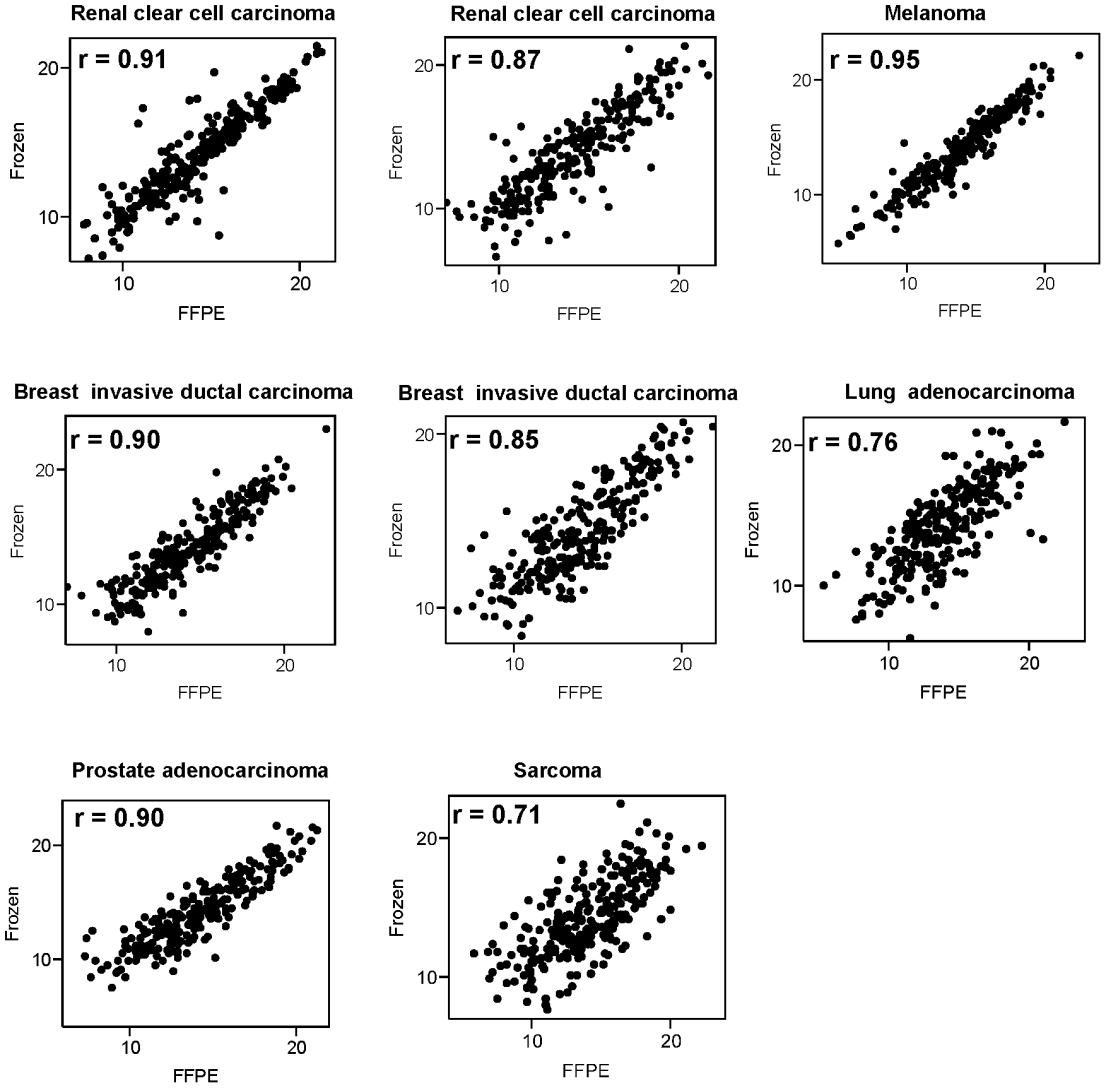
Scatter plots of detected microRNAs (250 miRs with no missing data) profiles obtained from matched fresh frozen-FFPE samples by SOLiD 4 system. Each dot represents the expression values of one microRNA in a fresh frozen-FFPE pair.

We next determined the correlation of microRNA expression values of matched FFPE samples and fresh frozen samples. There were 250 microRNAs detected in all samples in this study. There was a strong correlation of microRNA expression values in matched FFPE and snap fresh frozen tissues across all microRNAs and all cases (r  = 0.85; figure 6). Correlations across detected microRNAs from the same tumor/patient were high, with r ranging from 0.71–0.95 (figure 7) [25].

**Figure 8:**
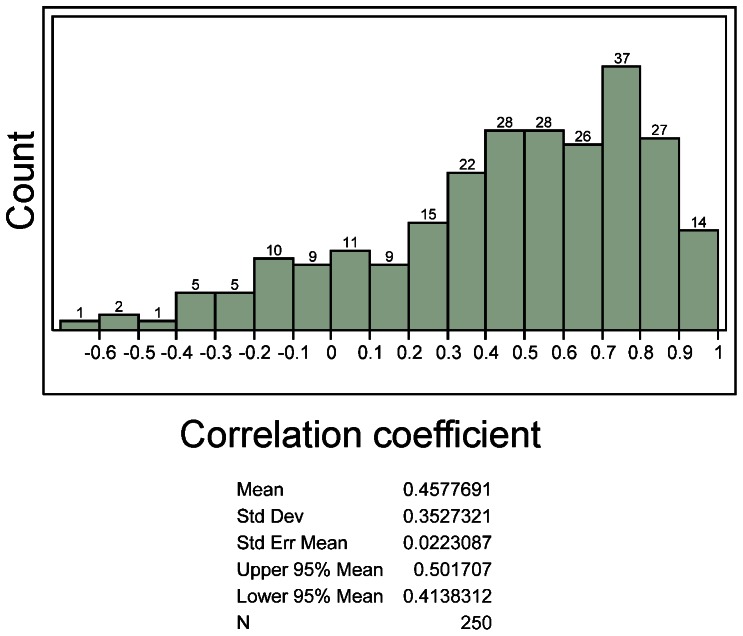
MicroRNA Pearson correlation coefficient distribution of eight matched fresh frozen and FFPE samples among the 250 microRNAs detected in all samples.

**Figure 9:**
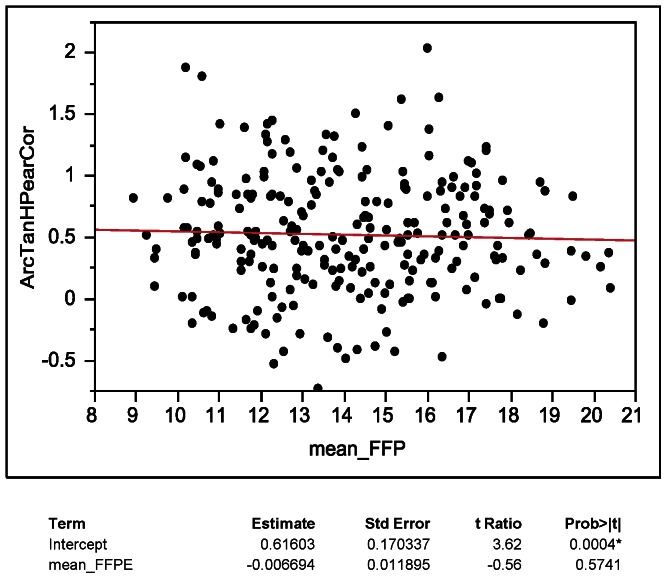
Scatter plot of microRNAs expression mean values versus Pearson correlation coefficients (Fisher transformed) from matched fresh frozen-FFPE samples.

**Figure 10:**
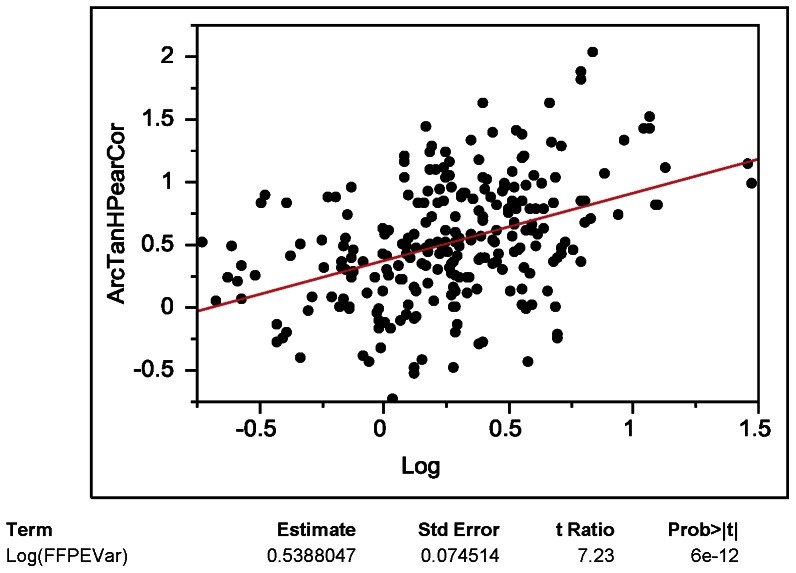
Scatter plot of microRNAs variance (log10 transformed) versus Pearson correlation coefficients (Fisher transformed) from matched fresh frozen-FFPE samples.

The mean of Pearson correlation coefficients of individual microRNA expression values of FFPE and frozen tissues was 0.46 (figure 8), with only 41 mature microRNAs and precursors having correlation coefficients higher than 0.8 (table 2). We sought out to identify features that are associated with higher correlation coefficients indicating invariance to the tissue preservation method. To test if the level of expression of a given microRNA is associated with the correlation between fresh frozen and FFPE tissue, Fisher transformed (arctanh) correlations were regressed onto mean FFPE expression (figure 9). There was no association between expression values and correlations between data from frozen and FFPE tissue observed. We then sought out to understand if a possible lack of variation of individual microRNAs among the biological replicates is resulting in poor correlations. Fisher transformed correlations were regressed onto log transformed FFPE variances. The variance analysis showed that microRNAs with higher variance have significantly higher correlations of data from FFPE and frozen tissues (figure 10; arctanh(r)  = 0.47+0.59×log10 (FFPE variance)), which may be due to the presence of true biological variation present in these microRNAs.

**Table 2:** 41 microRNAs had correlation coefficients equal to or greater than 0.8 between FFPE and fresh frozen pairs.

microRNA	Pearson correlation coefficient	Mature sequence	Genome coordinate
hsa-miR-199b-5p mature	0.96	CCCAGUGUUUAGACUAUCUGUUC	chr9: 131007000-131007109 [-]
hsa-mir-365-2 prec	0.94	UAAUGCCCCUAAAAAUCCUUAU	chr17: 29902430-29902540 [+]
hsa-mir-455 prec	0.94	GCAGUCCAUGGGCAUAUACAC	chr9: 116971714-116971809 [+]
hsa-mir-365-1 prec	0.93	UAAUGCCCCUAAAAAUCCUUAU	chr16: 14403142-14403228 [+]
hsa-mir-10a prec	0.93	UACCCUGUAGAUCCGAAUUUGUG	chr17: 46657200-46657309 [-]
hsa-miR-378*	0.93	CUCCUGACUCCAGGUCCUGUGU	chr5: 149112388-149112453 [+]
hsa-mir-143 prec	0.93	UGAGAUGAAGCACUGUAGCUC	chr5: 148808481-148808586 [+]
hsa-miR-199a-5p mature	0.92	CCCAGUGUUCAGACUACCUGUUC	chr19: 10928102-10928172 [-]
hsa-mir-199a-1 prec	0.92	CCCAGUGUUCAGACUACCUGUUC	chr19: 10928102-10928172 [-]
hsa-miR-148a mature	0.92	UCAGUGCACUACAGAACUUUGU	chr7: 25989539-25989606 [-]
hsa-mir-181b-1 prec	0.92	AACAUUCAUUGCUGUCGGUGGGU	chr1: 198828002-198828111 [-]
hsa-miR-204 mature	0.91	UUCCCUUUGUCAUCCUAUGCCU	chr9: 73424891-73425000 [-]
hsa-miR-182 mature	0.91	UUUGGCAAUGGUAGAACUCACACU	chr7: 129410223-129410332 [-]
hsa-mir-199a-2 prec	0.91	CCCAGUGUUCAGACUACCUGUUC	chr1: 172113675-172113784 [-]
hsa-mir-204 prec	0.9	UUCCCUUUGUCAUCCUAUGCCU	chr9: 73424891-73425000 [-]
hsa-mir-148a prec	0.9	UCAGUGCACUACAGAACUUUGU	chr7: 25989539-25989606 [-]
hsa-miR-130b mature	0.89	CAGUGCAAUGAUGAAAGGGCAU	chr22: 22007593-22007674 [+]
hsa-mir-100 prec	0.88	AACCCGUAGAUCCGAACUUGUG	chr11: 122022937-122023016 [-]
hsa-mir-125a prec	0.88	UCCCUGAGACCCUUUAACCUGUG	chr19: 52196507-52196592 [+]
hsa-mir-199b prec	0.87	GUACAGUAGUCUGCACAUUGGUUA	chr9: 131007000-131007109 [-]
hsa-mir-181b-2 prec	0.87	AACAUUCAUUGCUGUCGGUGGGU	chr9: 127455989-127456077 [+]
hsa-miR-424 mature	0.87	CAGCAGCAAUUCAUGUUUUGAA	chrX: 133680644-133680741 [-]
hsa-miR-27b mature	0.87	AGAGCUUAGCUGAUUGGUGAAC	chr9: 97847727-97847823 [+]
hsa-miR-30d mature	0.87	UGUAAACAUCCCCGACUGGAAGCU	chr8: 135817119-135817188 [-]
hsa-mir-10b prec	0.87	UACCCUGUAGAACCGAAUUUGUG	chr2: 177015031-177015140 [+]
hsa-mir-181a-2 prec	0.86	AACAUUCAACGCUGUCGGUGAGU	chr9: 127454721-127454830 [+]
hsa-mir-188 prec	0.86	CAUCCCUUGCAUGGUGGAGGG	chrX: 49768109-49768194 [+]
hsa-miR-660 mature	0.86	UACCCAUUGCAUAUCGGAGUUG	chrX: 49777849-49777945 [+]
hsa-mir-425 prec	0.85	AAUGACACGAUCACUCCCGUUGA	chr3: 49057581-49057667 [-]
hsa-mir-146a prec	0.84	UUGAGAACUGAAUUCCAUGGGU	chr5: 159912359-159912457 [+]
hsa-mir-146b prec	0.84	UGAGAACUGAAUUCCAUAGGCU	chr10: 104196269-104196341 [+]
hsa-let-7c prec	0.84	GGGUUGAGGUAGUAGGUUGUAUGGU	chr21: 17912148-17912231 [+]
hsa-mir-30d prec	0.83	UGUAAACAUCCCCGACUGGAAGCU	chr8: 135817119-135817188 [-]
hsa-let-7c mature	0.82	GGGUUGAGGUAGUAGGUUGUAUGGU	chr21: 17912148-17912231 [+]
hsa-let-7g*	0.82	CUGUACAGGCCACUGCCUUGC	chr3: 52302294-52302377 [-]
hsa-mir-328 prec	0.82	CCCCUGGCCCUCUCUGCCCUUCCG	chr16: 67236224-67236298 [-]
hsa-mir-181c prec	0.82	AACAUUCAACCUGUCGGUGAGU	chr19: 13985513-13985622 [+]
hsa-mir-182 prec	0.81	UUUGGCAAUGGUAGAACUCACACU	chr7: 129410223-129410332 [-]
hsa-miR-365 mature	0.81	UAAUGCCCCUAAAAAUCCUUAU	chr16: 14403142-14403228 [+]
hsa-miR-146a mature	0.81	UUGAGAACUGAAUUCCAUGGGU	chr5: 159912359-159912457 [+]
hsa-miR-22 mature	0.81	AAGCUGCCAGUUGAAGAACUGU	chr17: 1617197-1617281 [-]

### PCR validation of microRNA expression

**Figure 11:**
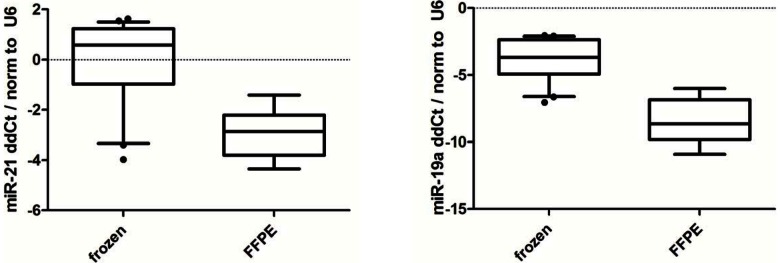
Validation of differentiated microRNA expression profile by RT-qPCR method. RNA samples from fresh frozen (n = 8) and FFPE (n = 8) tissues were analyzed. Small nuclear RNA U6 was used as reference. MiR-21 and miR-19a had higher expression in fresh frozen samples than in FFPE samples, consistent with sequencing results.

We selected two microRNAs (miR-21 and miR-19a) that were expressed higher in frozen samples than in FFPE samples in our microRNA sequencing results for validation using PCR (Taqman). Both miR-21 and miR-19a were found to also be higher expressed in frozen tissue than in matched FFPE samples using a PCR assay (figure 11), consistent with our sequencing results.

## Discussion

MicroRNAs as gene expression regulators appear to have an important role in cancer cell differentiation [4], proliferation [26], metastasis [27] and apoptosis [28]. There have been many microRNA profiling efforts using frozen cancer tissues [29] [30] [31]. Cancer tissue specimens are typically archived after formalin fixation and paraffin embedding (FFPE) and frozen tissue is only available for a minority of cases in pathology archives and tissue banks. A better understanding of the quality of microRNA sequencing results of FFPE tissues would allow for a more informed decision as to when use of FFPE tissue for small RNA sequencing experiments would be appropriate.

In this study, we analyzed small RNA sequencing profiles of eight pairs of matched fresh frozen and FFPE cancer tissue samples using the ABI SOLiD 4 platform. Storage time up to nine years appeared to not affect the number of detectable microRNAs in either frozen of FFPE tissue in this study. The presence of higher percentages of non-microRNAs in the fraction of nucleotides smaller than 40 nt suggested that FFPE tissues are more degraded, though. However, this apparent degradation did not result in fewer microRNAs being detected in FFPE tissues, suggesting that a sequencing approach can overcome sample degradation to some degree. While small RNAs in one of eight FFPE samples appeared degraded as suggested by the reduced read lengths centered at 17 nt compared to the other eight FFPE and all eight frozen samples centered at 21 nt, the number of aligned reads and the number of detected microRNAs in this degraded sample were similar to the data from other samples, which suggests that data obtained from samples with the above described degree of degradation would not necessarily need to be removed from all types of analysis.

Overall, there was excellent correlation between miRNA expression values between matched frozen and FFPE when combining all samples and all microRNAs (fig. 6), or when analyzing all microRNAs within individual patient sample pairs (fig. 7). Those findings are consisted with previous reports using different sequencing platforms suggesting that sequencing approaches can result in good correlations between FFPE and frozen samples when including several hundred microRNAs [32] [33] [9] [34].

Correlations of expression values of individual microRNAs (see figs. 8, 9, 10) were lower than correlations including all microRNAs (figs 6, 7). The underlying reason for this observation likely originates in the identified association of correlation coefficients of individual microRNAs and variance of microRNA expression values (fig 10). Very similar expression values of an individual microRNA among the eight cases (equal to low variance in fig. 10) typically result in low correlation coefficients because the variation between frozen and FFPE data is larger than the variation of expression between the eight cases included in this study. Therefore it is not necessarily surprising to have a wide distribution of correlations of individual microRNAs as analyzed in detail in figures 8, 9, and 10.

Limitations of this analysis include limited small sample size and potential PCR drifts and PCR selection biases during amplification in the multiplex PCR based library construction [35] [36]. This disadvantage should be overcome by the 3^rd^ generation of sequencing technology, which is thought to not require amplification of the target sequence anymore but detect single nucleic acid molecules [37]. Furthermore, the use of adjacent tissue to create matched specimens for this analysis introduces some tissue heterogeneity effects and likely contributes to the observed differences between matched frozen and FFPE samples. In summary, microRNA sequencing of FFPE tissues is feasible using ligation-based sequencing and microRNA expression values of individual patients are highly correlated with expression data from matched frozen tissues. MicroRNAs with higher variance appear to have a higher correlation of their expression values in matched frozen and FFPE tissue data.
